# Multifold pressure-induced increase of electric conductivity in LiFe_0.75_V_0.10_PO_4_ glass

**DOI:** 10.1038/s41598-019-53232-z

**Published:** 2019-11-12

**Authors:** Piotr Baranowski, Szymon Starzonek, Aleksandra Drozd-Rzoska, Sylwester J. Rzoska, Michal Bockowski, Pawel Keblinski, Tomasz K. Pietrzak, Jerzy E. Garbarczyk

**Affiliations:** 1 Faculty of Physics, Warsaw University of Technology, Warsaw, Poland; 2 Institute of High Pressure Physics of the Polish Academy of Sciences, Warsaw, Poland; 3 Materials Science and Engineering Department, Rensselaer Polytechnic Institute, Troy, NY USA

**Keywords:** Batteries, Structure of solids and liquids

## Abstract

We investigated the impact of high pressure and high-temperature annealing on lithium-vanadium-iron-phosphate (LiFe_0.75_V_0.10_PO_4_) glass materials, proposed for the use in cathodes for high-performance batteries. The treatment was carried out below the glass transition temperature (*T*_*g*_ ≈ 483 °C) at *P* = 1 GPa pressure, in an argon atmosphere. It led to the multifold electrical conductivity increase. Broadband dielectric spectroscopy (BDS) measurements before and after the process revealed the strong DC-conductivity increase across the whole studied frequency range by two orders of magnitude. The phenomenon is explained using Mott’s theory of polaron hopping in disordered solids containing transition metal oxides. The pressure evolution of the glass transition temperature and the crystallisation temperature above *T*_*g*_ is shown.

## Introduction

The change of the transportation systems towards electric cars fleet and inherently intermittent nature of many renewable energy sources, such as solar and wind electric power, requires breakthroughs in the development of innovative batteries characterised by a higher power and energy density matched with lower costs. Since lithium-iron-phosphate (LiFePO_4_) was indicated theoretically by Goodenough *et al*.^1^ to have a high electrochemical capacity, it became one of the most promising materials for Li-ion battery cathodes. However, its practical performance remains limited due to the relatively low electronic conductivity. This severe drawback has been addressed by numerous approaches, mainly based on carbon additives, structural modifications, and doping^2–4^. Whittingham *et al*.^5^ showed that small addition of vanadium has a positive impact on its gravimetric capacity. The following notable possibility offers the application of materials in the amorphous state instead of the crystalline one. The use of amorphous/glass material for electrodes results in isotropic properties avoids the detrimental role of grain-boundaries. Furthermore, glasses have ‘open structure’ with larger free volume, and they are characterised by the decoupling between ionic conduction and the structural mode relaxation^6,7^. To increase the electric conductivity of such glasses, Garbarczyk *et al*.^8,9^ developed the novel way of preparation of the electrodes, in which the increase of the electric conductivity is associated with the nucleation of nano-crystallites within the amorphous matrix. In particular, this method led to a giant (9 orders of magnitude) increase in electrical conductivity of LiFe_0.75_V_0.10_PO_4_ glass due to its thermal nano-crystallisation^10^. The nano-crystallisation was achieved by an exploration of the characteristic feature of glass-forming systems that exhibit a crystallisation zone in the ultraviscous domain above glass transition temperature *T*_*g*_, that emerges upon heating from the solid glass state^11,12^. In particular, rapid heating followed by a subsequent quench led to the formation of the composite system with LiFe_0.75_V_0.10_PO_4_ nano-crystallites within the residual solid amorphous matrix.

The mechanism of the observed significant increase of electric conductivity was explained within the classical Mott’s theory of electron (polaron) hopping in oxide glasses containing transition metal oxides (e.g., Fe and V)^13^, which was also adopted to nanomaterials^14^. In this approach, electrical conductivity is given by the formula^13,14^:

1 $$\sigma (T)={\nu }_{e}c(1-c)\frac{{e}^{2}}{R{k}_{B}T}\exp (-2\alpha R)\exp (-\frac{{E}_{a}}{{k}_{B}T})$$

where *R* is the average distance between hopping centres, *α* is the inverse localisation length of the electron wave function, *c* is the fraction of occupied hopping sites for electrons (e.g., Fe^2 +^ or V^4 +^ ions), and *E*_*a*_ is the activation energy of electronic conduction.

The activation energy can be expressed by^13,14^

2 $${E}_{a}=\frac{{e}^{2}}{16\,\pi {\varepsilon }_{0}{\varepsilon }_{p}{r}_{p}\,}(1-\frac{{r}_{p}}{R})+\frac{{W}_{D}}{2}$$

where *w*_*D*_ is the average difference in energy of the two hopping centres caused by a disorder (e.g., in glassy or nano-crystalline material), *ε*_*p*_ – relative electric permittivity of a polaron, *u*_*p*_ – its radius.

As indicating by Eqs (1) and (2) the composition and the structure of materials play a significant role in the electrical conductivity because they affect the average distance between hopping centres *R* and their concentration *c*. Furthermore, shells of nano-grains have a significant volumetric impact on the charge transfer process, due to strongly disordered interfacial regions which are characterised by higher densities of hopping centres. The disordered interfacial structure and small values of *R* might explain significantly higher values of electrical conductivity and lead to lower activation energies.

To investigate further the above-described hopping conductivity mechanism and the possibility of achieving even higher electrical conductivity increases, in this work we focus on the impact of compressing on electrical properties of LiFe_0.75_V_0.10_PO_4_ glasses and its nano-crystallite–glass composites.

In general, high pressures can lead to unique, and often exotic features^15–23^ such as the appearance of metallic hydrogen phase^20,21^ or polymeric nitrogen^22,23^. While these phenomena are scientifically fascinating, they often require pressures of the order hundreds of GPa, i.e., similar to those present in the centre of the Earth. The practical use of the pressure path pressure for the development of new materials has to address 3 essential problems: (i) pressures leading to significant effects are often within extreme, multi-GPa, domain, (ii) such extreme-pressures compression limits the number of transformed materials to few milligrams, (iii) unique properties disappear when returning to ambient conditions^15–23^. However, there is a notable exception to these limitations. Recently, it was shown that in the case of oxide glasses the high pressure (HP, ~1–2 GPa) and high temperature (HT, 600–800 °C) annealing at the adequately selected temperatures below the glass transition temperature *T*_*g*_ could permanently increase the glass density, the surface hardness and the cracking-resistance^24–26^.

Therefore, in this work, we examine the potential of combining the high-pressure and high-temperature annealing with the nano-crystallisation scheme recalled above for glassy LiFe_0.75_V_0.10_PO_4_ glasses.

## Results and Discussion

Glassy samples were synthesised from inorganic pre-dried precursors according to the routine described in detail in ref.^27^, providing melting in the inert atmosphere and fast cooling with melt-quenching technique. This process resulted in a reference glassy sample processed at atmospheric pressure (*P* = 0.1 MPa). Subsequently, such sample was pressurised at *P* = 1*GPa*, at a temperature just below *T*_*g*_(*P*), then it was fast heated up to *T*_*cryst*_(*P*) and later fast cooled down to *T*_*g*_(*P*) − 50 *K* where the sample was annealed for Δ*t* = 2*hours*. Finally, the sample was cooled to room temperature.

**Figure 1:**
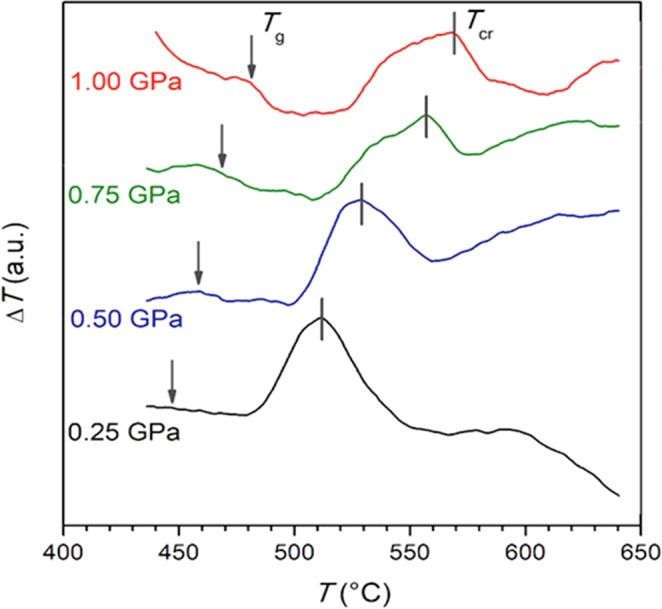
DTA traces of LiFe_0.75_V_0.10_PO_4_ glasses measured at different pressure. Studies were carried out under pressures indicated in the Figure.

For the differential thermal analysis (DTA, Fig. 1) under high pressure and temperature, glassy samples were placed into a graphite melting pot. During this process, the composite (amorphous matrix + nano-crystallites) appeared.

**Figure 2:**
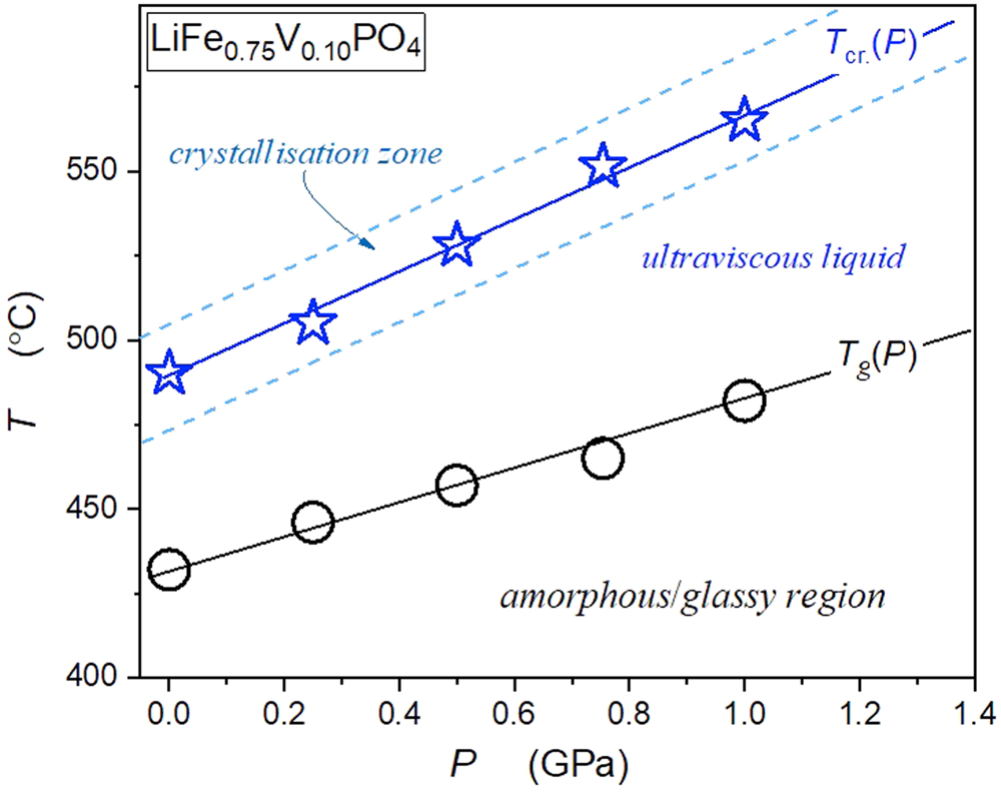
Pressure dependences of the glass transition temperature *T*_*g*_ (○) and the maximal crystallisation temperature *T*_*cr*_  for glass-forming LiFe_0.75_V_0.10_PO_4_. Dashed lines indicate the borders of the crystallisation zone.

Subsequently, high-pressure and high-temperature (HP-HT) studies were carried to reveal the glass transition temperature and the crystallisation domain dependence as the function of pressure. These results, obtained via the differential thermal analysis (DTA) under pressure, are shown in Fig. 2. The glass transition temperature and the maximal crystallisation follow the following dependencies: *T*_*g*_ = 57.5*P* + 432.5 (°C) and the temperature *T*_*cr*._78.5*P* + 488.5 (°C). Borders of the crystallisation zone are indicated in Fig. 2. The HP-HT annealing discussed below took into account conditions defined by Fig. 2, within the P-T plane.

**Figure 3:**
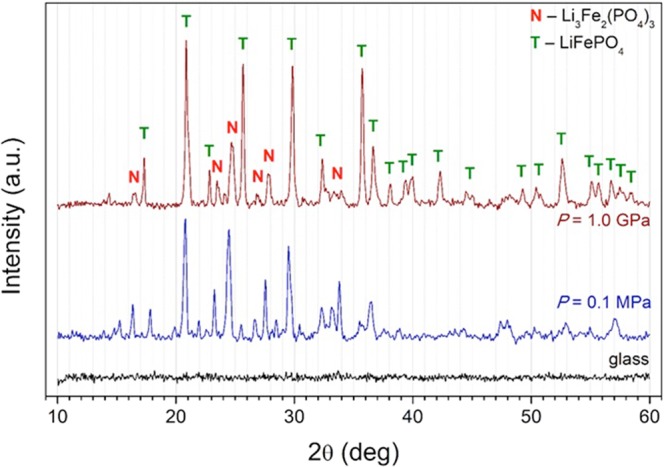
XRD ‘structural’ patterns of as-synthesised LiFe_0.75_V_0.10_PO_4_ glass (black line) and after its crystallisation under ambient pressure (blue line) and high pressure 1 GPa (dark red line). The diffraction peaks were ascribed to triphylite (*T*) and NASICON-like (*N*) phases.

Figure 3 shows the results of XRD analysis of samples after the temperature formation under atmospheric pressure and after the HP-HT annealing. One can conclude that nano-crystalline samples after pressurising exhibited a structure of lithium-iron-phosphate olivine with impurities of NASICON-like Li_3_Fe_2_(PO_4_)_3_ phase. The amount of the impurities was considerably lower in the sample heat-treated at the high-pressure *P* = 1 GPa. XRD results suggest that changes in the conductivity are caused by microscopic charge transfer mechanism based on electron hopping. These results are consistent with the activation energy comparison for both samples, presented below. According to Eq. (2) compressing can induce the increase of the electric conductivity, what is associated with the decrease of the activation energy, itself related to the decrease in an average distance between hopping centres (*R*).

**Figure 4:**
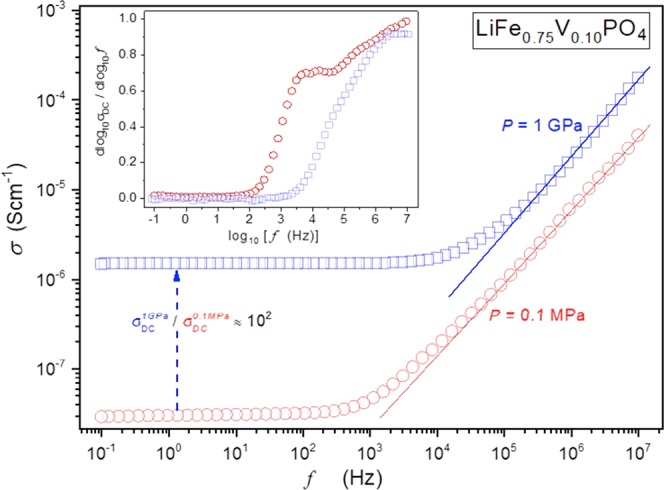
Electric conductivity as the function of frequency for LiFe_0.75_V_0.10_PO_4_ glassy composite system: the amorphous, glass matrix + nano-crystallites with the olivine structure at a temperature *T* = 100 °C. Results are for the thermal treatment at the atmospheric pressure *P* = 0.1 MPa  and for the pressure processing at *P* = 1 GPa , as described in the report. The horizontal part is for the DC electric conductivity. The inset shows the derivative of experimental data from the central part of the given figure, to show fine details of the behaviour.

The frequency dependences of the electric conductivity after the ‘standard’ thermal treatment under atmospheric pressure and after the HP-HT treatment are shown in Fig. 4. Both samples exhibit the ‘universal’ pattern of electric conductivity for ionic, conductive, systems^28,29^:

3 $$\sigma (f)={\sigma }_{DC}+{A}_{\sigma }{f}^{n}$$

where the horizontal low-frequency part is related to DC electric conductivity.

The analysis of the data in the power-law regions using the linear regression fit in the logarithmic scale part yielded exponents *n* ≈ 0.80 for *P* = 0.1*MPa* and *n* ≈ 0.88 as shown by blue and red lines. The low-frequency plateaus in Fig. 4 represent DC electric conductivity. Remarkably, there is a dramatic increase from ${\sigma }_{DC}^{0.1MPa}\approx 2.89\cdot {10}^{-8}$ S·cm^−1^ to ${\sigma }_{DC}^{1GPa}\approx 1.51\cdot {10}^{-6}$ S·cm^−1^ due to the above described HP-HT treatment. Moreover, the HP-HT treatment extends the DC electric conductivity domain. Its onset for the thermal treatment at *P* = 0.1 *MPa* is related to *f*_*on*_ ≈ 100 *Hz* where after the HP-HT treatment *f*_*on*_ ≈ 5 *kHz*.

The inset in Fig. 4 shows the derivative of experimental data from the central part of the plot, yielding the distortion-sensitive insight into its frequency dependence. The most notable features are that it reveals the significant violation of Josher’s ‘universal’ scaling^28,29^ relation for the frequency-dependent ‘DC part’ of electric conductivity, which according to Eq. (3) should manifest itself via the horizontal dependence with the value related to the exponent *n*. This means that the scaling’ behaviour in the main part of the plot should be treated instead as the ‘effective’ portrayal. Regarding the DC conductivity, the expected constant value of electric conductivity, i.e. *d log*_10_σ(*f*)/*d log f* = 0, occurs only for the HP-HT processed sample. For the samples, processed under atmospheric pressure, there is the small upward shift showing that is the case of the ‘almost-DC’ samples with ∼σ_*DC*_ ∝ *f*^1.02^.

**Figure 5:**
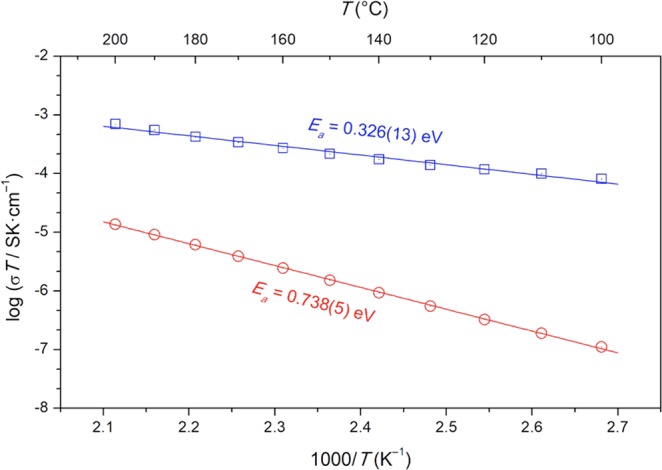
Temperature dependencies of electrical conductivity of nano-crystalline LiFe_0.75_V_0.10_PO_4_ samples measured at ambient pressure (red circles) and after the pressure processing at *P* = 1 GPa (blue squares).

Figure 5 shows the temperature evolution of the DC conductivity showing the clear Arrhenius type evolution:

4 $$\sigma (T)=\frac{{\sigma }_{0}}{T}\exp (-\frac{{E}_{a}}{kT})$$

where *E*_*a*_ stands for the activation energy.

Notable, that after HP-HT treatment, the activation energy is equal to 44% of the one observed the one noted due to the temperature treatment under atmospheric pressure.

Concluding, this report presents the first-ever pressure dependence of the glass transition temperature and the crystallisation temperature (T_*cr*._ > *T*_*g*_) for the Li-ion based glass formers. The new way of treatment of LiFe_0.75_V_0.10_PO_4_ glass matrix + nano-inclusions with olivine structure based on the HP-HT treatment led to the appearance of a set of favourable for applications features, in comparison with the standard treatment under atmospheric pressure. Notable is the range of applied temperatures and pressures, enabling the direct scaling at least to the pilot scale. All these indicate that the application of the properly designed high-pressure/high-temperature treatment can be considered as the new way in the challenge for innovative batteries solutions.

## Methods

We used Novocontrol Alpha Impedance Analyzer (model 2015 or broadband dielectric spectroscopy (BDS) measurements over the frequency range 10^−1^*Hz* < *f*10^7^*Hz*. Samples were placed between two flat-parallel capacitor’s plates made from Invar with a diameter 2*r* = 10 *mm* and a thickness *d* = 1 *mm* and using a measurement voltage *U* = 1*V*. The high-pressure (HP) and high-temperature (HT) processing were carried out using large-volume (V ~1 L) HP-HT facilities with gas nitrogen (N_2_) medium designed and built within IHHP PAS with a protocol described in refs^24–26^.
